# Attitude and Perception of Preclinical Undergraduate Medical Students about Problem Based Learning in Medical College of Nepal: A Descriptive Cross-sectional Study

**DOI:** 10.31729/jnma.8600

**Published:** 2024-06-30

**Authors:** Naresh Karki, Lokraj Joshi, Pravin Prasad, Kamal Kandel, Raju Prasad Shakya

**Affiliations:** 1 Department of Pharmacology, Lumbini Medical College and Teaching Hospital, Tansen, Palpa, Nepal; 2 Department of Physiology, Karnali Academy of Health Sciences, Jumla, Karnali, Nepal; 3 Department of Pharmacology, Institute of Medicine (TU), Maharajgunj, Kathmandu, Nepal; 4 Department of Pharmacology, Universal College of Medical Sciences and Teaching Hospital, Bhairahawa, Rupandehi, Nepal; 5 Department of Emergency Medicine, Lumbini Medical College and Teaching Hospital, Tansen, Palpa, Nepal

**Keywords:** *attitude*, *knowledge*, *perception*, *preclinical*, *problem based learning*

## Abstract

**Introduction:**

Problem-based learning is a student-centered learning method. Assessing students' understanding, settled way of thinking and interpretation towards problem-based learning is essential. The objective of the study was to understand the attitude and perception of preclinical M.B.B.S. students about problem-based learning.

**Methods:**

A descriptive cross-sectional study was conducted from July 5, 2023 to September 4, 2023 for the duration of two months after duly approval from Institutional Review Committee (Protocol No: IRC-LMC-04/Q-23). First and second year M.B.B.S. students who had good experience of attending problem-based learning sessions in integrated basic science subjects were included in the study. Fifteen close-ended questions related to attitude and perceptions were designed in Google Form. The responses were taken on five point Likert scale ranging from strongly disagree (1), disagree (2), neutral (3), agree (4), and strongly agree (5). Data were expressed as frequency and percentage.

**Results:**

Among 164 participants, 92 (56.09%) strongly agreed and 67 (40.87%) agreed that problem based learning enhances self-directed learning on the topic. Similarly, 103 (62.80%) strongly agreed and 59 (35.98%) agreed that problem based learning improves communication skills. Likewise, 78 (47.57%) agreed and 53 (32.32%) strongly agreed that this learning method improves confidence in decision-making. Moreover, 89 (54.28%) agreed and 58 (35.36%) strongly agreed that tutors motivate students to learn themselves through problem based learning.

**Conclusions:**

Majority of the students have positive attitude and good perception towards problem based learning. This method helps in generating skills like communication skills, group discussion, constructive critical reasoning, decision -making.

## INTRODUCTION

Problem-Based Learning (PBL) is an alternative method of learning in undergraduate medical (M.B.B.S.) curriculum that was first introduced in McMaster University of Canada in 1960s.^1,2^ This method is student-centered and always initiated with the introduction of problem based case scenario.^3-5^ In Nepal, many medical colleges are conducting PBL sessions during the preclinical phase of M.B.B.S.^6^

Several studies have shown that PBL is an important learning approach.^2-7^ There are numerous advantages of PBL like active participation of the students, improvisation of communication skills and promotion of self-directed learning.^4-8^ However, there are also few disadvantages like time consuming and students having lack of experience of it.^8-10^ Despite of having some reasonable advantages, PBL has been partially implemented in addition to lectures in many medical colleges of Nepal. Thus, assessing the student's understanding, settled way of thinking and interpretation towards PBL is helpful for understanding the effectiveness and usefulness of it in medical education.

Thus, the aim of the study is to assess attitude and perception of preclinical M.B.B.S. student about problem-based learning method.

## METHODS

A descriptive cross-sectional study was conducted in preclinical department of Lumbini Medical College and Teaching Hospital (LMCTH), Tansen, Palpa, Nepal for the duration of two months from July 5, 2023 to September 4, 2023. The study was started after duly approval from Institutional Review Committee of LMCTH (Protocol No: IRC-LMC-04/Q-23). An approval letter from the principal office of LMCTH was also taken to conduct the study among the students. Similarly, informed consents were also taken from the participants before the initiation of the study. All students of first and second year of M.B.B.S. who have learnt through PBL method in integrated basic science subjects irrespective of age and gender were included in the study. Since the participation in the study was based on voluntary basis and interest of the participants, so that those students who did not want to participate in the study were excluded. Likewise, those students who missed the majority of PBL sessions were also not included in the study.

Fifteen closed-ended questions were designed on five-point Likert scale from previous published study.^2,3^ as showed in Table 1. Likert scale was composed as strongly disagree (1), disagree (2), neutral (3), agree (4), and strongly agree (5). Higher responses were collectively considered as strongly agree and agree, while lower responses were collectively considered as strongly disagree and disagree. In addition, higher responses were represented as more positive attitude and good perception respectively. Further, a Google Form was also designed which included informed consent form, email address of participants, demographic profile of participants (gender, M.B.B.S. year) and the questions related to attitude and perception of PBL.

At the beginning, the students of first and second year of M.B.B.S. were oriented about the topic of the study, purpose of the study, students' role in the study and confidentiality of the participation. A pilot test was done among 15 students who fulfilled inclusion criteria to assess the clarity and reliability. Their attitude and perception was assessed by asking the same fifteen close-ended questions and using five-point Likert scale in Google form. The methods applied for pilot study was same as described earlier. The Cronbach's Alpha estimate was 0.82. Thus, the internal consistency of the study was found to be reliable for participants. For the content validation, the questionnaire was circulated to three PBL tutors with experience of conducting PBL sessions for at least five years and one biostatistician within the LMCTH. They independently rated each item of the questionnaire as satisfactory. Then, Google Forms consisted of fifteen close-ended questions were circulated to the participants using the online medium. Initially, participants were asked to read the informed consent form attached in Google Form under section one. Once the participants agreed to participate in the study, they were asked to enter into section two to complete the questions of the study. After completion of all the questions in Google Forms, the participants were asked to submit them. All the participants were entered into the study consecutively and conveniently. Confidentiality of the participants was ensured by de-identifying the data in records. The data were also secured by password protected software.

Data of the participants were exported from Google Form to MS Excel and then into Statistical Package for Social Sciences (SPSS). Data were expressed as frequency and percentage.

## RESULTS

Out of 200 total students of first year and second year of M.B.B.S., 164 (82%) responses were received. Of them, 83 (50.60%) were female and 83 (50.60%) were belonged to second year of M.B.B.S. Among 164 participants, 92 (56.09%) strongly agreed and 67 (40.87%) agreed that problem based learning enhances self-directed learning on the topic. Similarly, 103 (62.80%) strongly agreed and 59 (35.98%) agreed that problem based learning improves communication skills. Likewise, 78 (47.57%) agreed and 53 (32.32%) strongly agreed that PBL improves confidence in decision-making.

**Table 1:** Attitude and perception of students towards problem-based learning (n = 164).

SN.	Questions	1	2	3	4	5
		Strongly Disagree	Disagree	Neutral	Agree	Strongly Agree
		n (%)	n (%)	n (%)	n (%)	n (%)
1	PBL passes on the knowledge of the content of the topic better than traditional lecture	2 (1.22)	5 (3.05)	29 (17.69)	86 (52.44)	42 (25.60)
2	PBL retains the knowledge better than lectures do	-	6 (3.66)	30 (18.30)	81 (49.40)	47 (28.64)
3	PBL enhances self-directed learning on the topic	1 (0.60)	1 (0.60)	3 (1.84)	67 (40.87)	92 (56.09)
4	PBL makes the topic more interesting to learn than lectures do	1 (0.60)	6 (3.66)	24 (14.64)	75 (45.74)	58 (35.36)
5	PBL promotes exploration of different learning materials	-	6 (3.66)	19 (11.58)	85 (51.83)	54 (32.93)
6	PBL improves communication skills	-	-	2 (1.22)	59 (35.98)	103 (62.80)
7	PBL improves teamwork abilities	-	3 (1.83)	20 (12.20)	72 (43.90)	69 (42.07)
8	PBL makes better interaction between students	-	1 (0.61)	6 (3.66)	80 (48.78)	77 (46.95)
9	PBL makes better interaction between teacher and student	-	5 (3.05)	20 (12.20)	69 (42.07)	70 (42.68)
10	PBL improves confidence in decision-making	1 (0.61)	2 (1.22)	30 (18.29)	78 (47.56)	53 (32.32)
11	PBL helps to identify strengths and weaknesses of students	1 (0.61)	7 (4.27)	23 (14.02)	81 (49.39)	52 (31.71)
12	Tutors motivate students to learn themselves through PBL	1 (0.61)	3 (1.83)	13 (7.93)	89 (54.27)	58 (35.37)
13	PBL should be added with traditional curriculum	2 (1.22)	8 (4.88)	27 (16.46)	74 (45.12)	53 (32.32)
14	More PBL sessions should be included in each year	-	11 (6.71)	40 (24.39)	56 (34.15)	56 (34.15)
15	The tutors of LMCTH are well prepared to facilitate PBL	5 (3.05)	14 (8.54)	37 (22.56)	69 (42.07)	39 (23.78)

Moreover, 89 (54.28%) agreed and 58 (35.36%) strongly agreed that tutors motivate students to learn themselves through problem based learning. In addition, 69 (42.07%) agreed and 39 (23.79%) strongly agreed that tutors of Lumbini Medical College and Teaching Hospital are well prepared to facilitate PBL. (Table 1) Furthermore, 125 (76.21%) of the total students use the text books and reference books as preferred resource material during problem based learning as showed in Table 2. The overall ratings given by the students for PBL as learning method were 48 (29.26%) excellent, 77 (46.95%) very good, 33 (20.13%) good and 6 (3.66%) fair.

**Table 2:** Preferred places and use of resources for self-directed learning (n= 164).

Variables	n (%)
Preferred places for self-directed learning	
Library	12 (7.32)
Home	61 (37.19)
Both library and home	87 (53.05)
Others	4 (2.44)
Resources for self-directed learning	
Text books and Reference books	125 (76.21)
Internet	34 (20.74)
Class Notes	5 (3.05)

## DISCUSSION

This study was done to understand the attitude and perception of preclinical M.B.B.S. students towards problem-based learning method. An attitude is a positive, negative or mixed assessment of an object expressed at some level of intensity. While, perception is the organization, recognition and analysis of sensory information in order to represent and perceive the presented information. Majority of the students participated in this study positively and in good manner have stated that problem-based learning method enhances self-directed learning, encourages active participation of students, makes the topics more interesting to learn, improves communication and decision making skills. Many of them also stated that tutors of LMCTH are well prepared to conduct problem-based learning.

In this study, nearly 90% of the participated students admitted that problem-based learning enhances self-directed learning on the topic. This finding was similar to the other studies conducted on different settings nationally and internationally.^5-8^ Problem-based learning enhances self-directed learning on the topic. Self-directed learning is the self-guided study where the students take the initiative themselves for learning activities with or without helps from others.

Students plan and manage the study hours to find out the learning objectives, learning resources and learning outcomes. Then, they search, read and gather appropriate knowledge and information on the learning topics from the learning resources like text books, reference books and internet. Further, they share and discuss their knowledge with the teammates during next group gathering of same problem-based learning session. The role of the teacher in selfdirected learning is only providing advises, directions, resources and materials.^9,10^

Similar to other studies, the majority of the students in our study acknowledged that problem based learning makes the topic more interesting to learn than lectures do.^7,11^ It might be because of clinical scenario of the problem. The problem is generally written in storytelling manner. Students discuss the problem which briefly contains clinical history of a patient, clinical examinations, laboratory investigation findings and treatment of the disease. Some other features that make the problem-based learning sessions more interesting are active participation of students, problem-solving tasks and communication between the students.^11,12^ Beside this, students also share their knowledge and experiences in the group that make the session interactive, attentive and curious.

Moreover, in this study students perceived that problem-based learning improves communication skills which was comparable with other studies.^3,4,10^ One of the advantages of conducting problem-based learning is developing communication skill because all the students in a group are encouraged to participate actively in every task like group discussion and presentation. The tutors also motivate every student in the group to communicate and perform the task. Communication skill is important for medical students while studying or once they enter the medical profession. For students, communication ability is necessary during history taking, conducting clinical examinations, presentations in class and community, and attending practical examinations. Likewise, for doctors communication skill is important during consultation with patients, attending the conferences, seminars, and workshops. Similarly, preclinical and clinical academicians with better communication skills can develop good explaining ability to deliver the lecture and can engage the students in the classroom.

Decision making skill is perceived as determining single best option from many choices to accomplish the target. About three-fourth of total students in our study have a settled thinking that problem based learning improves decision making skill which were supported by other studies conducted in the past.^2,4^ Problem-based learning is architected mainly to boost the problem solving ability, critically thinking and creative skills. It may help students to understand their abilities and talents, to identify various available choices and to choose one option during decision making. Thus, problem-based learning may improve learners' higher order of thinking skills and develops confidence in decision making during real life problem situations.

Approximately, two-third of the participants in our study interpret that tutors are well prepared to conduct the problem based learning sessions. Here, the well prepared tutors mean that they are doing their minimal roles during the problem-based learning sessions like guidance, resource providing, group dynamics and evaluation of students. This finding was supported by some studies.^6,8,14^ The role of the tutors in problem-based learning is making conducive environment for learning process, guiding the students if students are going out of the track, motivating each student for active participation in the study, providing the learning resources / materials and evaluation of knowledge and skills of students.^12,13,15^ There are some factors that affect the tutor performance like lack of knowledge and skills of conducting problem-based learning, lack of experience of it, lack of experience of constructing case, leniency and stringency.^12,13^ For conducting problem-based learning sessions effectively, teachers should be enthusiastic and have ability to engage the students in the group. The regular training of tutors is an essential tool that will help them lot to update the knowledge and skills of running problem-based learning efficiently.^13,14^

One important limitation of the study was that the selfreported responses were provided by the students rather than gathering the responses directly and objectively. Besides, the other limitation was that the study was conducted in small number of participants. However, this study will help to conduct other similar types of studies in future among larger numbers of students and medical colleges.

## CONCLUSIONS

The majority of the students have good perception and positive attitude towards problem based learning. This method helps in gaining the desired knowledge as well as in generating the skills like communication skills, group discussion, constructive critical reasoning and decision-making.
